# Optimal cut-offs of five anthropometric indices and their predictive ability of type 2 diabetes in a nationally representative Kenyan study

**DOI:** 10.3934/publichealth.2021041

**Published:** 2021-07-09

**Authors:** Anthony Muchai Manyara

**Affiliations:** Social and Political Sciences, Institute of Health and Wellbeing, University of Glasgow, Glasgow, UK

**Keywords:** diabetes, prediction, anthropometric cut-offs, Kenya, Africa, waist circumference, waist-to-height ratio

## Abstract

**Background:**

Type 2 diabetes (T2D) is one of the top non-communicable diseases in Kenya and prevention strategies are urgently needed. Intervening to reduce obesity is the most common prevention strategy. However, black populations develop T2D at lower obesity levels and it is unclear which anthropometric cut-offs could provide the best predictive ability for T2D risk. This study, therefore, aimed to determine the optimal anthropometric cut-offs and their predictive ability of T2D in Kenya.

**Methods:**

The study included 2159 participants (59% women) aged 35–70 years from the Kenya STEPwise survey conducted in 2014. Five anthropometric indices were used—body mass index (BMI), waist circumference (WC), waist to hip ratio (WHR), waist to height ratio (WHtR) and waist divided by height^0.5^(WHt.5R). Diabetes was defined as a fasting blood glucose of ≥7.0 mmol/l or a previous diagnosis by a health worker. Optimal anthropometric cut-offs and their receiver operating characteristics, such as the area under the curve (AUC), were computed.

**Results:**

Overall, the optimal cut-off for BMI, WC, WHR, WHtR and WHt.5R were 24.8 kg.m^−2^, 90 cm, 0.88, 0.54 and 6.9. On disaggregation by sex, the optimal cut-off for BMI, WC, WHR WHtR and WHt.5R was 27.1 kg.m^−2^, 87 cm, 0.85, 0.55 and 6.9 in women, and 24.8 kg.m^−2^, 91 cm, 0.88, 0.54 and 6.9 in men. Overall, WC (AUC 0.71 (95% confidence interval 0.65, 0.76)) WHtR (AUC 0.71 (0.66, 0.76)) and WHt.5R (AUC 0.70 (0.65,0.75)) had a better predictive ability for T2D than BMI (AUC 0.68 (0.62, 0.73)).

**Conclusions:**

WC, WHtR and WHt.5R were better predictors of T2D than BMI and should be used for risk stratification in Kenya. A WC cut-off of 87cm in women and 91cm in men, a WHtR cut-off of 0.54 or a WHt.5R of 6.9 in both men and women should be used to identify individuals at an elevated risk of T2D.

## 1. Introduction

The prevalence of diabetes is on the rise in sub-Saharan Africa (SSA) exerting a disease and economic burden [1]. Type 2 diabetes (T2D), the main form of diabetes, is preventable through lifestyle modification (e.g. weight loss) [2]. Excess body fat is an important risk factor for T2D and anthropometric indices that estimate body fat, such as body mass index (BMI), waist circumference (WC), waist to hip ratio (WHR), and waist to height ratio (WHtR), have been used to predict risk [3]. However, debate remains on which is the best index to stratify risk. Despite BMI being the most used index it has been challenged on its inability to: 1) differentiate between body lean mass and fat mass; and 2) detect the location of body fat [4]. On the other hand, central obesity indices (WC, WHR, WHtR), that are proxies of visceral fat, may be better measures of risk as visceral fat is more important for T2D development than peripheral fat [5]. Consequently, a meta-analysis of 20 studies reported that WHtR and WC provide a more robust measure for detecting T2D than BMI [6]. Apart from the four common anthropometric indices, new indices have recently been proposed. The waist divided by height^0.5^ (WHt.5R) has been found to be a better predictor of cardiometabolic risk than BMI, WC, WHR, and WHtR [7] although it is relatively understudied.

In SSA, several studies have reported that central obesity indices are better predictors of T2D than BMI [8]–[14]. However, it remains unclear which index has a better predictive ability. For instance, in Ghana and Ethiopia, WHR was a better predictor of T2D in three studies [9],[12],[13] while WC was a better predictor of T2D in five studies from Ethiopia, Cameroon, Kenya, and Guinea [8],[10]–[13]. Further, there is increasing evidence that black populations develop T2D at lower obesity levels compared to whites, and therefore current anthropometric cut-offs may be less appropriate for them. For example, the age-adjusted prevalence of T2D in whites at 30 kg.m^−2^, WC of 102 cm in men, and 88 cm in women equated to a similar prevalence at a BMI of 26 kg.m^−2^, WC of 88 cm and 79 cm in men and women respectively in the black populations in the UK [15]. There is emerging but limited evidence of the optimal cut-offs to predict T2D in SSA. In Ghana, the optimal cut-offs for BMI, WC and WHR were 26.2 kg.m^−2^, 91.7 cm and 0.88 for women, and 26.7 kg.m^−2^, 83.4 cm and 0.90 for men [9]. Recently, an Ethiopian study reported that the optimal cut-offs for BMI, WC, WHR and WHtR were 20.5 kg.m^−2^, 82.9 cm, 0.86 and 0.51 in women, and 23.0 kg.m^−2^, 88.6 cm, 0.97 and 0.52 in men [13]. The optimal anthropometric cut-offs and their predictive ability of T2D in Kenya remain understudied. Therefore, this study aimed to determine the optimal anthropometric cut-offs of five indices (BMI, WC, WHR, WHtR, WHt.5R) for predicting T2D in Kenya and compare their predictive ability.

## 2. Materials and methods

The study used data from the Kenya STEPwise survey 2014 which was a nationally representative study that investigated non-communicable diseases risk factors. A multi-stage cluster sampling method was used to recruit 4500 participants aged 18–70 years. Data were collected by trained personnel and involved the administration of a questionnaire, physical and biological measurements [16]. More information on the survey can be found elsewhere [16].

### 2.1. Inclusion and exclusion criteria

**Figure 1.**
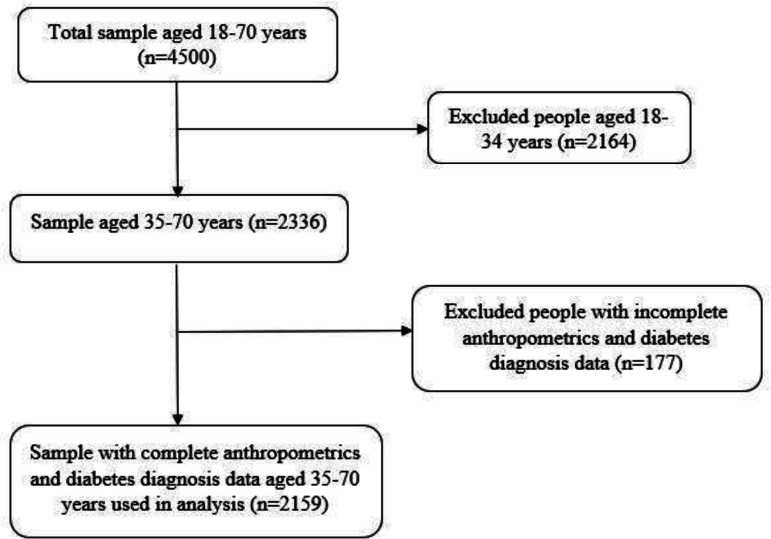
Inclusion and exclusion of study participants.

Figure 1 shows the inclusion and exclusion of study participants. To limit analyses to type 2 diabetes cases, we included participants between 35 and 70 years of age as type 2 diabetes prevalence increases with age [17] and type 1 diabetes is relatively low from ≥35 years of age [18]. A total of 2159 participants with complete data on all anthropometric measures and diabetes diagnosis were included in the analysis.

### 2.2. Data collection procedures and definitions

Height was measured in centimetres using a portable stadiometer and weight was measured in kilogram using a portable electronic weighing scale. Waist circumference was measured at the umbilicus level using a constant tension tape. Blood pressure was measured three times using an automated blood pressure measuring instrument (OMRON®). A minimally invasive prick was used to draw a blood sample and CardiocheckPA analyser® was used to measure blood glucose levels [16]. Body mass index was calculated by dividing weight in kilograms by height in metres squared. It was categorised into: underweight (<18.5 kg.m^−2^), normal weight (18.5–24.9 kg.m^−2^), overweight (25–29.9 kg.m^−2^) and obese (≥30 kg.m^−2^) [19]. WHR was calculated by dividing waist circumference by hip circumference in centimetres. WHtR was calculated by dividing waist circumference by height in centimetres. WHt.5R was calculated by dividing waist circumference by height in centimetres raised to the power of 0.5. Diabetes was defined as a fasting glucose of ≥7.0 mmol/l or a previous diagnosis by a health worker [20].

### 2.3. Ethics approval and consent to participate

The study was approved by the Kenya Medical Research Institute's Ethics Review Committee (SSC No. 2607). Informed written consent was sought from all participants before data collection.

### 2.4. Analysis

All data analysis was done using R statistical package version 3.5.2 [21]. Means and standard deviations were used to summarise continuous variables, and frequencies and percentages used to summarise categorical variables. The Analysis of Variance and the Chi-Square tests were used to assess significant sex differences in continuous and categorical variables, respectively. Associations between T2D and anthropometric indices were determined using logistic regression models and odds ratios (with 95% confidence intervals). Model 1 was unadjusted and model 2 was adjusted for age, residence (rural/urban), education level, wealth index and systolic blood pressure. The anthropometric indices were standardised in the models to determine the effect of one standard deviation increase on odds of T2D. The ‘OptimalCutpoints' package was used to compute the optimal anthropometric cut-offs and their receiver operating characteristics (ROC) using the Youden Index. The characteristics computed were: the Area Under the Curve (AUC), sensitivity, specificity, positive predictive value, and negative predictive value associated with each cut-off [22]. The ‘pROC’ package was used to compare the AUC of anthropometric measures using BMI as the reference [23]. The ROC curves of all five anthropometric indices were plotted to show sensitivity (y-axis) and specificity (x-axis). Data are presented for the overall sample and by sex disaggregation. A statistical significance level of p < 0.05 was used.

## 3. Results

Table 1 presents the characteristics of the 2159 study participants included in the analyses. The average age was 48 years, and the diabetes prevalence was higher in women (6.5%) compared to men (4.3%). The majority of both women and men were of normal weight status.

Table 2 shows the associations between anthropometric indices and T2D. Each standard deviation increase in all the five anthropometric indices was associated with a higher T2D risk in both the overall sample and in women in model 2. In men, BMI, WHR and WHtR were not associated with T2D but each standard deviation increases in WC and WHt.5R were associated with higher odds of T2D in model 2.

**Table 1.** Characteristics of study participants disaggregated by sex.

	All (n = 2159)	Women (n = 1271)	Men (n = 888)	P value (Women vs. Men)
Age [mean ± SD]	48.1 ± 9.9	48.4 ± 10.0	47.7 ± 9.8	0.118
Residence
Rural	1196 (55.4)	755 (59.4)	441 (49.7)	**<0.001**
Urban	963 (44.6)	516 (40.6)	447 (50.3)	
Education level
No formal schooling	452 (20.9)	348 (27.4)	104 (11.7)	**<0.001**
Primary school incomplete	569 (26.4)	354 (27.9)	215 (24.2)	
Primary school complete	645 (29.9)	373 (29.3)	272 (30.6)	
Secondary school and above	493 (22.8)	196 (15.4)	297 (33.4)	
Wealth index
1 (Poorest)	464 (21.5)	296 (23.3)	168 (18.9)	**<0.001**
2	477 (22.1)	283 (22.3)	194 (21.8)	
3	493 (22.8)	314 (24.7)	179 (20.2)	
4	393 (18.2)	219 (17.2)	174 (19.6)	
5 (Richest)	332 (15.4)	159 (12.5)	173 (19.5)	
Height(cm) [mean ± SD]	163.6 ± 9.5	159.2 ± 7.8	170.1 ± 8.0	**<0.001**
Diabetes prevalence [n (%)]	121 (5.6)	83 (6.5)	38 (4.3)	**0.032**
Systolic blood pressure (mmHg) [mean ± SD]	134.0 ± 23.5	134.1 ± 24.4	133.8 ± 22.2	0.790
Diastolic blood pressure (mmHg) [mean ± SD]	85.8 ± 13.4	86.6 ± 13.7	84.7 ± 13.0	**0.001**
BMI (kg·m^−2^) [mean ± SD]	24.2 ± 5.9	25.2 ± 6.2	22.7 ± 5.0	**<0.001**
Weight status [n (%)]
Underweight	240 (11.1)	109 (8.6)	131 (14.8)	**0.026**
Normal	1108 (51.3)	587 (46.2)	521 (58.7)	
Overweight	491 (22.7)	325 (25.6)	166 (18.7)	
Obese	320 (14.8)	250 (19.7)	70 (7.9)	
Hip circumference (cm) [mean ± SD]	95.8 ± 14.6	97.9 ± 15.7	92.7 ± 12.4	**<0.001**
Waist circumference (cm) [mean ± SD]	82.6 ± 14.7	83.1 ± 15.3	81.9 ± 13.8	0.058
WHR [mean ± SD]	0.86 ± 0.08	0.85 ± 0.08	0.88 ± 0.08	**<0.001**
WHtR [mean ± SD]	0.51 ± 0.09	0.52 ± 0.10	0.48 ± 0.08	**<0.001**
WHt.5R [mean ± SD]	6.5 ± 1.2	6.6 ± 1.2	6.3 ± 1.0	**<0.001**

Note: P values in bold are significant. BMI—body mass index; WHR—waist to hip ratio; WHtR—waist to height ratio; WHt.5R—waist divided by height0.5; SD—standard deviation.

**Table 2.** Associations between anthropometric indices and T2D in overall sample, women, and men.

	Model 1		Model 2	
	Odds ratio (95% CI)	P value	Odds ratio (95% CI)	P value
All
BMI	1.45 (1.25, 1.67)	**<0.001**	1.32 (1.13, 1.53)	**<0.001**
WC	1.95 (1.62, 2.35)	**<0.001**	1.62 (1.32, 1.98)	**<0.001**
WHR	1.54 (1.30, 1.83)	**<0.001**	1.36 (1.13, 1.63)	**0.001**
WHtR	1.88 (1.58, 2.24)	**<0.001**	1.58 (1.31, 1.91)	**<0.001**
WHt.5R	1.96 (1.63, 2.36)	**<0.001**	1.62 (1.33, 1.98)	**<0.001**
Women
BMI	1.46 (1.21, 1.75)	**<0.001**	1.29 (1.07, 1.56)	**0.009**
WC	2.02 (1.60, 2.56)	**<0.001**	1.62 (1.25, 2.08)	**<0.001**
WHR	1.75 (1.41, 2.18)	**<0.001**	1.56 (1.24, 1.96)	**<0.001**
WHtR	1.93 (1.54, 2.42)	**<0.001**	1.53 (1.20, 1.95)	**<0.001**
WHt.5R	2.01 (1.59, 2.54)	**<0.001**	1.59 (1.24, 2.05)	**<0.001**
Men
BMI	1.33 (1.05, 1.68)	**0.018**	1.15 (0.85, 1.56)	0.364
WC	1.79 (1.31, 2.43)	**<0.001**	1.48 (1.03, 2.13)	**0.035**
WHR	1.36 (1.02, 1.83)	**0.038**	1.18 (0.84, 1.64)	0.342
WHtR	1.66 (1.24, 2.23)	**<0.001**	1.37 (0.97, 1.94)	0.076
WHt.5R	1.76 (1.29, 2.39	**<0.001**	1.44 (1.00, 2.08)	**0.048**

Note: Values are odds ratios (95% confidence intervals) based on a standard deviation increase in the anthropometric index. Bold p-values are significant. BMI—body mass index; WC—waist circumference; WHR—waist to hip ratio; WHtR—waist to height ratio; WHt.5R—waist divided by height^0.5^. Model 1 is unadjusted, and Model 2 is adjusted for age, wealth index, education level, residence (rural/urban), and systolic blood pressure.

**Figure 2.**
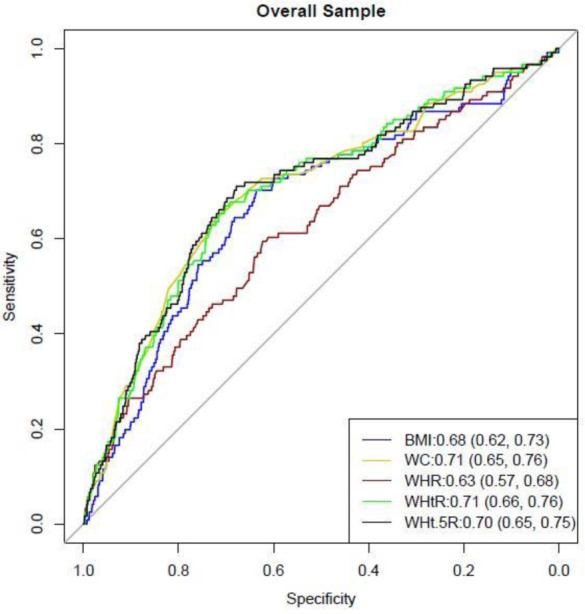
Receiver Operating Characteristic analyses for diabetes by anthropometric indices in the overall sample. The Area Under the Curve (95% confidence intervals) is shown inside the graph. Significant difference in Area Under the Curve were: p = 0.031 for BMI versus WC, p = 0.018 for BMI versus WHtR, and p = 0.022 for BMI versus WHt.5R.

**Figure 3.**
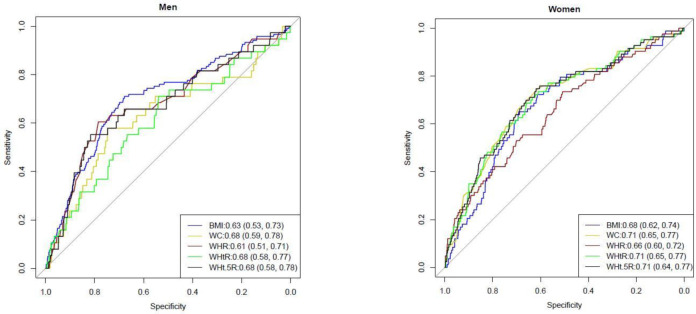
Receiver Operating Characteristic analyses for diabetes by anthropometric indices in women and men. The Area Under the Curve (95% confidence intervals) is shown inside the graphs. Significant difference in Area Under the Curve were: p = 0.021 for BMI versus WC, p = 0.032 for BMI versus WHtR, p = 0.018 for BMI versus WHt.5R in men.

Figures 2 and 3 present the ROC analyses for T2D by anthropometric indices while Table 3 presents the optimal cut-offs of anthropometric indices and their predictive ability of T2D. Overall, the optimal cut-offs for BMI, WC, WHR, WHtR and WHt.5R were 24.8 kg.m^−2^, 90 cm, 0.88, 0.54 and 6.9. Overall, T2D was correctly discriminated by WC and WHtR 71% of the time (i.e., mean AUC was 0.71 in both) and 70% of the time by WHt.5R compared to 68% of the time by BMI, p = 0.031 for BMI versus WC, p = 0.018 for BMI versus WHtR, and p = 0.022 for BMI versus WHt.5R.

The optimal cut-offs for BMI, WC, WHR, WHtR and WHt.5R in women were 27.1 kg.m^−2^, 87 cm, 0.85, 0.55 and 6.9. In women, WC, WHtR and WHt.5R (AUC 0.71 in all) had a 3% better discrimination for T2D than BMI (AUC 0.68) although these differences did not reach significance level, p = 0.074 for BMI versus WC, p = 0.099 for BMI versus WHtR, and p = 0.075 for BMI versus WHt.5R. The optimal cut-offs for BMI, WC, WHR, WHtR and WHt.5R in men were 24.8 kg.m^−2^, 91 cm, 0.88, 0.54 and 6.9. In men, WC, WHtR and WHt.5R (AUC 0.68 in all) had a 5% better discriminative ability for T2D than BMI (AUC 0.63), p = 0.021 for BMI versus WC, p = 0.032 for BMI versus WHtR, p = 0.018 for BMI versus WHt.5R.

**Table 3.** Optimal anthropometric cut-offs and their predictive ability of diabetes in overall sample, women, and men.

	Optimal cut-off	AUC (95% CI)	P value difference in AUC	Sensitivity (%)	Specificity (%)	Positive predictive value (%)	Negative predictive value (%)
Body mass index (kg.m^−2^)							
Overall	24.8	0.68 (0.62, 0.73)	Reference	70 (62, 78)	64 (62, 66)	11 (10, 16)	97 (96, 97)
Women	27.1	0.68 (0.62, 0.74)		65 (54, 76)	70 (67, 72)	14 (12, 21)	96 (94, 97)
Men	24.8	0.63 (0.53, 0.73)		58 (41, 74)	75 (72, 78)	10 (8, 18)	97 (95, 98)
Waist circumference (cm)							
Overall	90.0	0.71 (0.65, 0.76)	**0.031**	65 (56, 73)	73 (71, 75)	13 (12, 18)	97 (96, 97)
Women	87.0	0.71 (0.65, 0.77)	0.074	75 (64, 84)	62 (60, 65)	13 (12, 20)	97 (95, 97)
Men	91.0	0.68 (0.59, 0.78)	**0.021**	61 (43, 76)	79 (76, 82)	12 (10, 22)	98 (96, 98)
Waist to hip ratio							
Overall	0.88	0.63 (0.57, 0.68)	0.133	60 (51, 69)	63 (61, 65)	9 (8, 13)	96 (95, 97)
Women	0.85	0.66 (0.60, 0.72)	0.512	74 (63, 83)	51 (48, 54)	10 (9, 16)	96 (94, 97)
Men	0.88	0.61 (0.51, 0.71)	0.726	71 (54, 85)	54 (50, 57)	7 (6, 14)	98 (95, 98)
Waist to height ratio							
Overall	0.54	0.71 (0.66, 0.76)	**0.018**	67 (58, 75)	71 (69, 73)	13 (12, 18)	97 (96, 97)
Women	0.55	0.71 (0.65, 0.77)	0.099	71 (61, 81)	65 (62, 67)	13 (12, 20)	97 (95, 97)
Men	0.54	0.68 (0.58, 0.77)	**0.032**	55 (38, 71)	82 (79, 85)	13 (11, 23)	98 (95, 98)
Waist divided by height^0.5^							
Overall	6.9	0.70 (0.65, 0.75)	**0.022**	71 (62, 79)	68 (66, 70)	12 (11, 17)	98 (96, 98)
Women	6.9	0.71 (0.64, 0.77)	0.075	75 (64, 84)	62 (59, 65)	12 (11, 19)	97 (95, 98)
Men	6.9	0.68 (0.58, 0.78)	**0.018**	63 (46, 78)	78 (75, 81)	11 (10, 21)	98 (96, 98)

Note: P values in bold are significant. AUC—Area Under the Curve; CI—confidence interval. Data in brackets are 95% confidence intervals. P values for differences in AUC use BMI as the reference

## 4. Discussion and conclusions

This study aimed to determine optimal anthropometric cut-offs to predict T2D and compare their predictive ability in Kenya. The optimal BMI cut-off was 27.1 kg.m^−2^ in women and 24.8 kg.m^−2^ in men. These findings were consistent with a Ghanaian study which found an overweight BMI of 26.2 kg.m^−2^ as the optimal cut-off in women [9] and an Ethiopian study that reported a normal weight BMI of 23.0 kg.m^−2^ as the optimal cut-off in men [13]. Also, this is in agreement with increasing evidence that black populations develop T2D at a lower BMI [24] than the current obesity cut-off of 30 kg.m^−2^ [25]. Further, WC and WHtR had a better discriminatory ability for T2D than BMI. This agrees with other findings from Guinea, Kenya, Cameroon, and Ethiopia which have shown that WC is a better predictor of T2D [8],[10]–[13]. Globally, these findings are consistent with a meta-analysis that found that WC and WHtR had better discrimination for T2D than BMI [6]. WC and WHtR are proxies of visceral fat which confers a higher risk of metabolic complications such as T2D compared to peripheral fat [26]. This implies that these measures, rather than BMI, should be used for risk stratification in Kenya.

We found the optimal WC cut-off as 87 cm for women and 91 cm for men. These cut-offs differ from those from other SSA settings: in Ghana, the optimal cut-off was 91.7 cm for women and 83.4 cm for men [9] while in Ethiopia the optimal cut-off was 82.9 cm for women and 88.6cm for men [13]. Despite these contrasts, our findings show a similar trend to the other two studies, that is, the cut-offs for women are above the WHO cut-off for metabolic complications (≥80 cm) while the cut-offs in men are below the WHO thresholds (≥94 cm) [25]. A similar trend has been reported by a large analysis of data (n = 24181) from eight SSA countries which showed that men were at increased cardiometabolic risk at lower WC (≥81.2 cm) than current guidelines (≥94 cm) [27]. This implies that a WC cut-off of ≥91 cm should be used for predicting T2D risk in men instead of the recommended ≥94 cm. In women, the WHO recommended cut-off of ≥88 cm which corresponds to “substantially increased risk” of metabolic complications [25] should be used to predict T2D risk in Kenya.

The WHt.5R was not found to be significantly better than the WHtR in predicting T2D. The optimal WHtR cut-off was relatively similar for women and men:0.55 and 0.54, respectively. This is slightly higher than what was reported in Ethiopia:0.51 in men and 0.52 in women [13]. The derived WHtR cut-offs are relatively similar to the recommended and widely used cut-off of ≥0.5 in women and men which translates to a simple screening message of “keep your waist to less than half your height” [28]–[30]. Apart from a simple public health message, the same WHtR cut-off is used for both men and women, and adults and children [30]. Further, WHtR is easier to measure and calculate than BMI and a string can be used for measurement (a string measuring half someone's height should fit around their waist) making it a very cheap screening method [28],[30]. This implies there is need to consider the use of WHtR in risk stratification and associated simple public health messaging to intervene on central obesity and prediction of T2D risk in Kenya.

Finally, the optimal anthropometric cut-offs in this study were lower than those reported among black populations in high-income countries. For example, we found the optimal BMI cut-off as 24.8kg.m^−2^ which is lower than what has been reported in black populations in the UK: 28.1kg.m^−2^ [31]. Similarly, recent evidence from the Research on Obesity and Diabetes among African Migrants (RODAM) study found that the optimal anthropometric cut-offs were highest in migrant Ghanaians in Europe and lowest in Ghanaians in rural Ghana [32]. Such variations in the optimal cut-offs could be due to a less central role of obesity in T2D development, higher risk even with modest increases in obesity, or increased significance of other risk factors. More research is needed to understand the mechanisms responsible for these variations in black populations.

This study adds to the limited evidence on optimal anthropometric cut-offs and their predictive ability of T2D in Kenya and SSA. Its strengths are the use of a large nationally representative sample, and identification of optimal cut-offs, and predictive ability of five anthropometric indices. However, just like the recommended cut-offs, the study uses cross-sectional evidence which limits causal inference of derived cut-offs on T2D. More evidence, particularly from prospective studies, is needed to confirm if the derived cut-offs are the most appropriate for T2D prediction in Kenya.

In conclusion, WC, WHtR and WHt.5R were better predictors of T2D than BMI highlighting their utility in risk stratification. The optimal cut-offs of WC (87 cm for women and 91 cm for men), WHtR (0.54 for both women and men) and WHt.5R (6.9 for both women and men) should be used for risk stratification in Kenya.
